# Manganese in Drinking Water: Bouchard Responds

**DOI:** 10.1289/ehp.1103485R

**Published:** 2011-06

**Authors:** Maryse F. Bouchard

**Affiliations:** Department of Environmental and Occupational Health, University of Montreal, Montreal, Québec, Canada, E-mail: maryse.bouchard@umontreal.ca

Chen and Copes raise some interesting issues regarding our article (Bouchard et al. 2011). In our study we investigated the change in IQ scores with respect to different exposure metrics for manganese. One of these metrics was home tap water manganese concentration, which was strongly associated with IQ deficits. Chen and Copes indicate that they consider it inappropriate to include in this analysis children who did not drink tap water at home. Second, they note that even for children in the highest quintile of water manganese concentration, the intake of manganese from water ingestion is below the recommended dietary manganese intake (Institute of Medicine 2001). In response to their first point, it is important to consider that children who do not drink tap water are still exposed through the consumption of many foods and drinks prepared with tap water. In addition, and perhaps most important, children might be exposed by inhalation of aerosols containing manganese when showering (Elsner and Spangler 2005). If this represents a significant source of exposure, which is unclear (Aschner 2006; Spangler and Elsner 2006), inhalation of aerosols could be responsible for inducing neurotoxic effects. Indeed, inhaled manganese is delivered to the brain much more efficiently than ingested manganese, because it bypasses normal homeostatic mechanisms.

Third, Chen and Copes make the point that because dietary intake of manganese intake is much higher than the amount ingested from water, the decrease in IQ is more likely due to intake of manganese from water and food sources collectively, rather than from water alone. The intake of manganese from water consumption was indeed very small compared with dietary intake (medians, 8 and 2,335 μg/kg/month, respectively), but we found no evidence that dietary manganese is related to cognitive abilities. As we reported in our article (Bouchard et al. 2011), dietary manganese intake, assessed with a food frequency questionnaire, was not associated with IQ and did change the point estimates for water manganese concentration when included in the regression model.

We believe that the interpretations that assimilate manganese present in water to dietary manganese have had the effect of dismissing the potential risks of this source of exposure, thus slowing research into this question. Little is known about the absorption and retention of manganese from food versus water, or about inhalation of aerosols in showers. Although more research is necessary to understand the mechanisms by which manganese present in water might be neurotoxic for children, we believe that our findings offer strong support for this hypothesis. Because manganese levels associated with significant cognitive deficits in our study are common in groundwater, this problem could have a great public health importance. For instance, 11% of domestic wells have manganese concentrations > 140 μg/L in the United States (U.S. Geological Survey 2009). We agree that additional studies, ideally with a prospective design, are necessary.

Finally, a valid biomarker of manganese exposure would greatly advance our understanding of this metal’s toxic effects. We used hair, notably because its collection is much less invasive than blood sampling. Chen and Copes rightly point out the limitations of hair as a biomarker, and research should explore new biomarkers. For instance, in a small study, saliva manganese levels were significantly higher in welders than in nonexposed subjects, and levels increased in welders with the more years of exposure (Wang et al. 2008). Also in that study, saliva manganese concentrations correlated with serum concentrations. Saliva is less invasive to collect than blood and less prone to external contamination than hair; thus, it might be a useful biomarker.
